# Novel DMAP@Mesoporous Silica Hybrid Heterogeneous Catalysts for the Knoevenagel Condensation: Greener Synthesis through Eco‐friendly Solvents

**DOI:** 10.1002/cplu.202400741

**Published:** 2025-03-01

**Authors:** Julio C. Fernandes P. Brito, Fabio Travagin, Mauro Barbero, Cristina Esteban, Urbano Díaz, Alexandra Velty, Giovanni B. Giovenzana, Ivana Miletto, Enrica Gianotti

**Affiliations:** ^1^ Dipartimento per lo Sviluppo Sostenibile e la Transizione Ecologica Università del Piemonte Orientale Piazza Sant'Eusebio 5 I-13100 Vercelli Italy; ^2^ Dipartimento di Scienze del Farmaco Università del Piemonte Orientale Largo Donegani 2 I-28100 Novara Italy; ^3^ Instituto de Tecnología Química Universitat Politècnica de València-Consejo Superior de Investigaciones Científicas Avenida de los Naranjos s/n E-46022 Valencia Spain

**Keywords:** Green grafting, Hybrid Catalyst, Nucleophilic Catalysis, DMAP

## Abstract

This article reports a sustainable synthesis of a novel organic‐inorganic hybrid catalysts, featuring 4‐(dimethylamino)pyridine (DMAP) immobilized onto mesoporous MCM‐41 silica and amorphous Aerosil silica supports. Using (±)‐2‐methyltetrahydrofuran (MeTHF), a bio‐based solvent, the covalent binding of DMAP to silica surfaces was achieved, reducing reliance on traditional petroleum‐based solvents like toluene. The DMAP‐functionalized hybrid catalysts, characterized through XRPD, TGA/DTA, FE‐SEM, and FT‐IR, demonstrated effective catalytic performance in the Knoevenagel condensation, a reaction relevant in producing fine chemicals and pharmaceuticals. The mesoporous MCM‐41‐supported catalyst exhibited superior activity due to its high surface area and ordered porous structure, with 97 % yield and 99 % selectivity. Stability and reusability were validated through leaching and recycling tests, confirming minimal DMAP leaching and robust catalytic performance over consecutive cycles. This green synthetic pathway underscores the potential of hybrid catalysts in advancing sustainable chemistry, promoting reduced energy consumption, and supporting a circular economy through recyclable, highly active catalysts in eco‐friendly solvents. These findings demonstrate that MCM‐41‐supported DMAP hybrids are viable candidates for eco‐friendly applications.

## Graphical abstract

This study presents a sustainable approach to functionalizing DMAP by introducing an aminomethyl group for stable anchoring on mesoporous silica supports, creating an effective, recyclable hybrid catalyst. Characterized by techniques like XRD, TGA, and FT‐IR, the hybrid retained stability and showed strong catalytic performance for the Knoevenagel reaction, highlighting its eco‐friendly application in green chemistry.

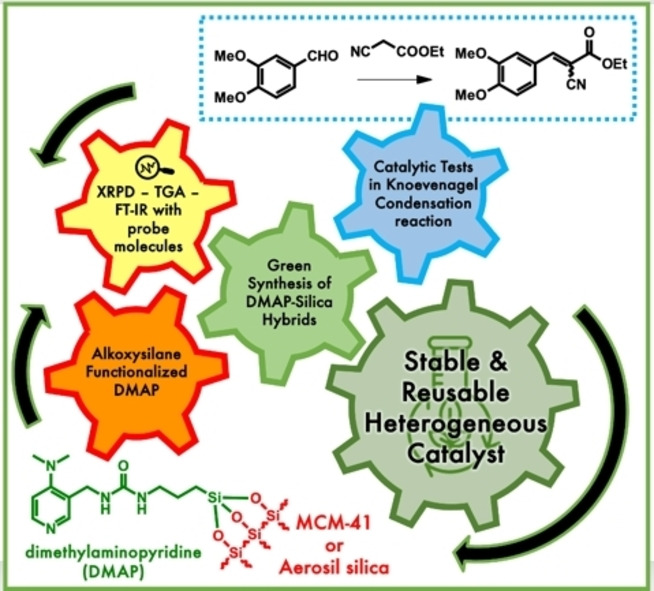

## Introduction

The development of sustainable chemical syntheses and processes is a priority task to reduce the utilization of fossil resources and to mitigate climate change. The use of heterogeneous catalysts to enhance the efficiency of a chemical reaction (less energy‐consuming) is nowadays a common strategy to match the principles of Green Chemistry.

In this context, organic‐inorganic hybrids are successfully employed as heterogeneous catalysts as they combine the versatility of the organic functionalities with the robustness and thermal stability of inorganic supports, that allows to stabilize and protect the organic moieties. Hybrid organic‐inorganic catalysts may be used in mild reaction conditions, enhancing energy efficiency, process efficiency and economic viability, while also reducing the carbon footprint, thereby fulfilling some of key objectives of green and circular chemistry.

Among the various hybrids that can be synthesized, heterogeneous nucleophilic catalysts are of particular interest in the chemical industry for the preparation of APIs, fine chemicals and polymers.[^1^, ^2^, ^3^, ^4^, ^5^] 4‐(Dimethylamino)pyridine (DMAP) is one of the most used nucleophilic, acid‐base and acyl transfer organocatalyst, being extremely efficient towards different organic transformations and reasonably cheap.[^6^, ^7^, ^8^, ^9^] Nevertheless, its significant toxicity profile makes it mandatory to avoid DMAP contamination of the reaction products and avoiding its release in the environment.

To this purpose, covalent immobilization of the catalyst onto a support to obtain a robust supported catalyst is a common and useful strategy to allow its recovery and recycling, with an overall positive impact on environment, process efficiency and economic viability. Different organic polymers have been employed as supports for DMAP with good results in the catalysis of esterifications, silylations and Baylis‐Hilmann reactions.^[10]^ Surprisingly, only few examples of immobilization of DMAP on inorganic support to give heterogeneous hybrid catalysts have been explored to date.[^11^, ^12^, ^13^]

In this work, ordered mesoporous MCM‐41 silica nanoparticles with high surface area has been used as support to heterogenized DMAP and compared with an amorphous silica (Aerosil) support.

A novel functionalization of DMAP has been developed, involving the innovative introduction on the pyridine ring of an aminomethyl group as a viaticum to its heterogenization. The newly implanted functional group has been employed for the covalent grafting of DMAP molecules onto the silanol groups present on the internal surface of mesoporous MCM‐41 and of Aerosil.

Due to the increasing importance of organic‐inorganic hybrids in catalysis, the synthetic approach to these functional materials is claiming an increasing attention of the scientific community, with the aim to improve the overall sustainability of their preparation and use. These hybrid materials can be used as heterogeneous catalysts when the organic functionality is bound, through a covalent bond (Class II hybrids), to an inorganic support.[^14^, ^15^, ^16^, ^17^, ^18^] The most common strategy to link by a covalent bond the organic functionality and the surface groups of the inorganic support is the post‐synthetic grafting, requiring the use of solvents, generally toluene, to bring into solution the organic reactants and to disperse the support particles. As the solvent represents a larger portion of the reaction mass, the possibility of substituting petroleum‐derived solvents for renewable ones in the procedures of material synthesis is of paramount importance to reduce the environmental impact of this reaction (and in general, for all reactions).

(±)‐2‐Methyltetrahydrofuran (MeTHF) is a readily available, inexpensive and biobased solvent derived from the catalytic hydrogenation of furfural, obtained from food industry wastes.[^19^, ^20^] MeTHF is an aprotic solvent with a boiling point of 78 °C and forming a useful azeotrope with water (10.6 % H_2_O). Its favourable solvent properties have been successfully exploited in the preparation of DMAP‐silica hybrid catalysts of this work.

The synthesized hybrid catalysts have been fully characterized with a multi‐technique approach (XRPD, TGA/DTA, FE‐SEM, N_2_ adsorption/desorption at 77 K, and FT‐IR spectroscopy using CO_2_ as acid probe) and used as a heterogeneous catalyst for Knoevenagel condensation to produce substituted ethyl 2‐cyano‐3‐phenylacrylate derivatives under environmentally friendly reaction conditions and microwave heating. The Knoevenagel reaction is a powerful tool in organic synthesis for the formation of a wide array of valuable compounds. The reaction between aromatic aldehydes and ethyl cyanoacetate exemplifies its value in generating a variety of products with potential applications in pharmaceuticals, food additives, agrochemicals and plastics. Moreover, microwave heating offer some benefits over conventional heating, such as higher rate of reaction, better yield and higher selectivity (less formation of side product), high reproducibility due to the uniformity of microwave irradiation and finally a lower energy consumption.

## Results and Discussion

Conventional approaches for anchoring catalytic moieties to silica surface often involve pre‐functionalizing the inorganic support with an alkoxysilane derivative. This introduces a functional group that can react with a reactive derivative of the catalytic moiety to be anchored. Despite being widely exploited, this approach has significant drawbacks. It is unlikely that the functional groups introduced on the inorganic support will be completely consumed after anchoring the catalytic moiety. Consequently, the final hybrid will expose both the catalytic moiety and the unreacted functional group of the alkoxysilane derivative, which can somewhat interfere with the adsorption/desorption of reactant and product and/or with the catalytic activity. In our approach, an alkoxysilane derivative of DMAP was synthesized and grafted on silica.

Functionalization of DMAP with a reactive moiety is a viaticum to its heterogenization. The DMAP molecule is not easily functionalized as such: the limited reactivities of the pyridine ring towards aromatic electrophilic substitution and of the tertiary amine in position 4 collide with the strong reactivity of the annular nitrogen atom.

Common approaches reported to date to heterogenized DMAP rely uniquely on the amino group in position 4 as the preferred linking point for a small and flexible aliphatic chain bearing a reactive alkoxysilane residue^[11]^ or for an alkyne residue for click‐chemistry based grafting.^[13]^ Such functionalized DMAPs has to be synthesized *ex novo*, usually by starting from 4‐methylaminopyridine^[11]^ or 4‐chloropyridine.^[21]^

Direct functionalization of the DMAP nucleus may offer a robust alternative platform for its heterogenization, provided that the troublesome reactivity of DMAP is suitably tackled. Even if pyridine is known to be quite unreactive towards aromatic electrophilic substitution, the presence of the 4‐dimethylamino group may be of help to overcome the scarce proclivity of the heteroaromatic nucleus to the attack of incoming electrophiles, usually intercepted by the annular nitrogen atom. A strong electrophile must be used to force the DMAP nucleus to react, and the choice fell on the phthalimidomethyl cation generated in the Tscherniak‐Einhorn amidomethylation.^[22]^

**Scheme 1:**
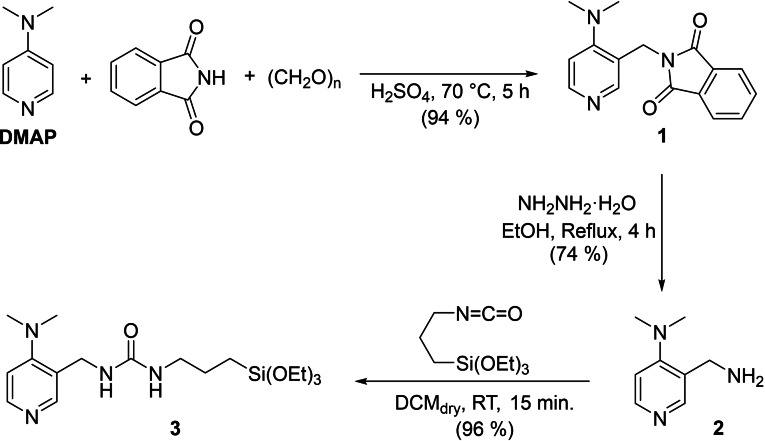
Synthesis of silylated DMAP (DMAP−Si).

The one‐pot Tscherniak‐Einhorn reaction was applied for the first time to DMAP, using *N*‐hydroxymethylphthalimide (generated *in situ* by the reaction of phthalimide and paraformaldehyde) as the electrophile precursor. The reaction was performed in concentrated sulfuric acid at 70 °C for 5 h (Scheme 1). Pouring the reaction mixture in water at pH higher than 10, led to the precipitation of the pure 3‐aminoalkylated derivative (**1**), isolated by filtration in 94 % yield.

The phthalimide group was removed by treatment with hydrazine hydrate in refluxing ethanol for 4 h, giving, after workup, 3‐(aminomethyl)‐4‐(*N,N*‐dimethylamino)pyridine (**2**) in 74 % yield (Scheme 1). We exploited the primary aliphatic amino group by reacting compound **2** with 3‐triethoxysilylpropyl isocyanate to give the grafting reagent **3**. The latter plays the role of the linker and embodies the trialkoxysilyl group, routinely employed for the grafting to the surface silanols of the silica thereof.

To covalently bind DMAP−Si on the silanol groups of the silica supports, MeTHF was used as an efficient and green alternative solvent to toluene. MCM‐41 NPs and silica Aerosil were treated with compound **3** (10 % w/w) in MeTHF and in toluene, for comparison, in the same conditions (60 °C, 24 h); after filtration and oven drying, a solid powder was obtained. In Table 1, the acronyms, the solvents used for the DMAP grafting procedure and the type of inorganic supports are listed.

**Table 1:** Acronyms of the DMAP hybrid materials.

Acronym	Solvent used for DMAP grafting	Inorganic Support	Loading
Hyb‐1	MeTHF	MCM‐41 NPs	10 % w/w
Hyb‐2	MeTHF	Aerosil	10 % w/w
Hyb‐3	toluene	MCM‐41 NPs	10 % w/w
Hyb‐4	toluene	Aerosil	10 % w/w

**Scheme 2:**
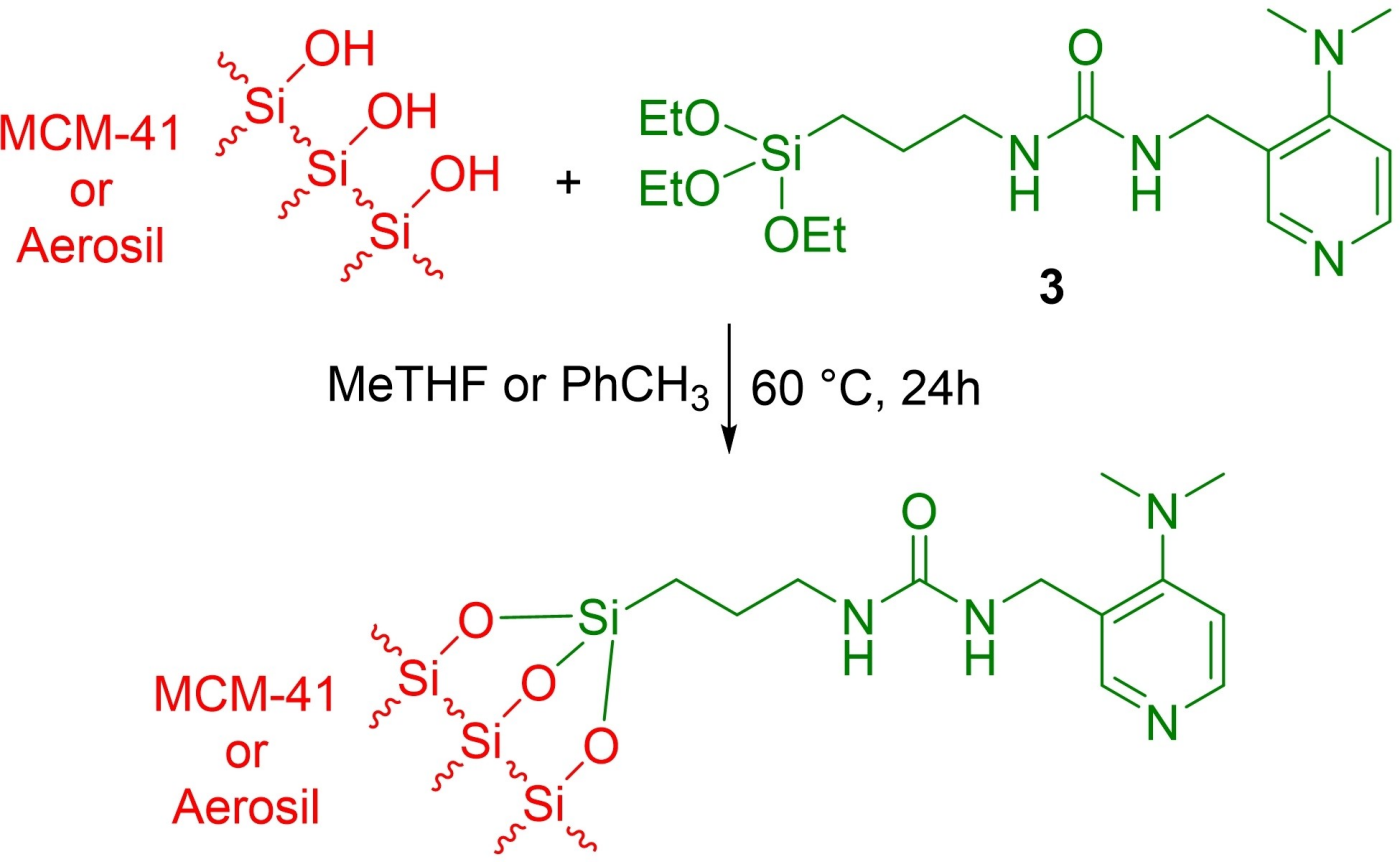
Immobilization of DMAP−Si on silica supports.

The organic‐inorganic hybrid catalysts based on the silylated‐DMAP grafted on the surface Si‐OH groups of the mesoporous silica nanoparticles have been obtained using a green grafting procedure substituting toluene with MeTHF as solvent (Scheme 2). Upon organic functionalization, some structural properties have been evaluated; in particular, the maintenance of the ordered mesoporous structure of MCM‐41 NPs was monitored by XRPD analysis (Figure S10 in the ESI). The low‐angle XRD patterns of Hyb‐1 reveal the presence of three reflections centered at 2θ=2.4°, 3.8° and 4.8° assigned to (100), (110) and (200) planes, pointing out that the hexagonal symmetry of MCM‐41 is maintained after grafting. The volumetric analysis of the Hyb‐1 exhibited IV‐type isotherm typical of mesoporous materials with a specific surface area of ca. 620 m^2^ gr^−1^ and a pore distribution centered at ca. 33 Å (Figure S11 in the ESI).

Both hybrids exhibited an aggregated morphology, as evidenced by FE‐SEM imaging (Figure S12 of the ESI).

**Figure 1:**
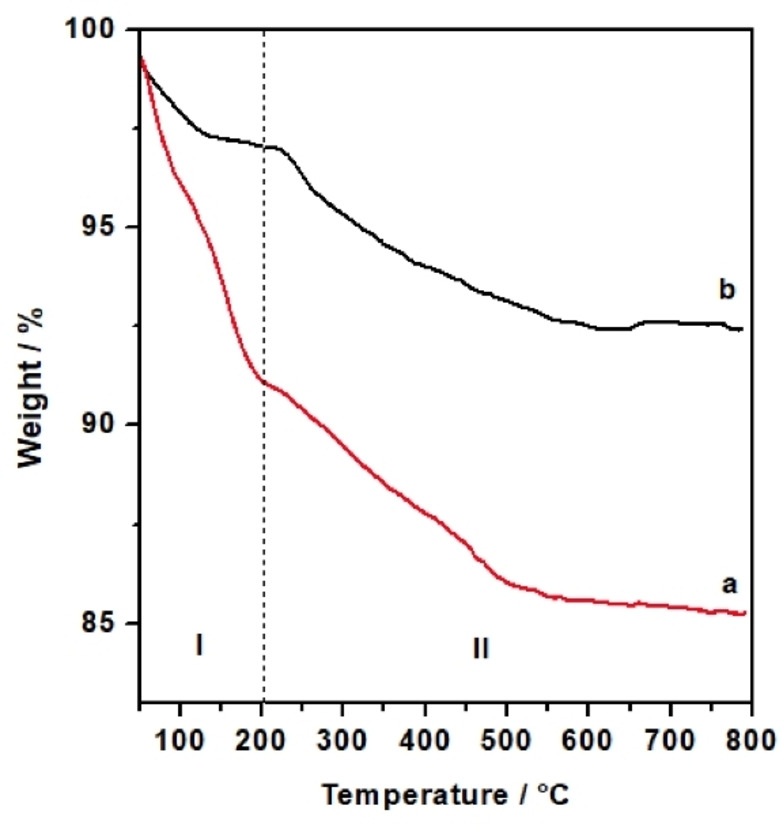
TGA analysis of Hyb‐1 (curve a, red) and Hyb‐2 (curve b, black).

To gain insight on the organic content presents in the hybrid solids and on the thermal stability of the inserted DMAP units, thermogravimetric analysis was performed (Figure 1 and Figure S13 in the ESI).

The first weight loss, observed at temperature lower than 200 °C (zone I), is associated to the removal of physisorbed water. The higher weight loss % of Hyb‐1 with respect to Hyb‐2 revealed a higher hydrophilic character of ordered mesoporous silica, also associated to a higher specific surface area. At higher temperature, the main weight loss is associated to the decomposition of the organic moieties (DMAP−Si) and the organic content calculated between 200 °C and 800 °C is similar in both hybrids (Table 2).

**Table 2:** Weight loss (Δwt %) calculated from TGA analysis.

Sample	Range I (<200 °C): Δwt (%)	Range II (200‐800 °C): Δwt (%)
Hyb‐1	9.05	5.49
Hyb‐2	3.01	4.53

**Figure 2:**
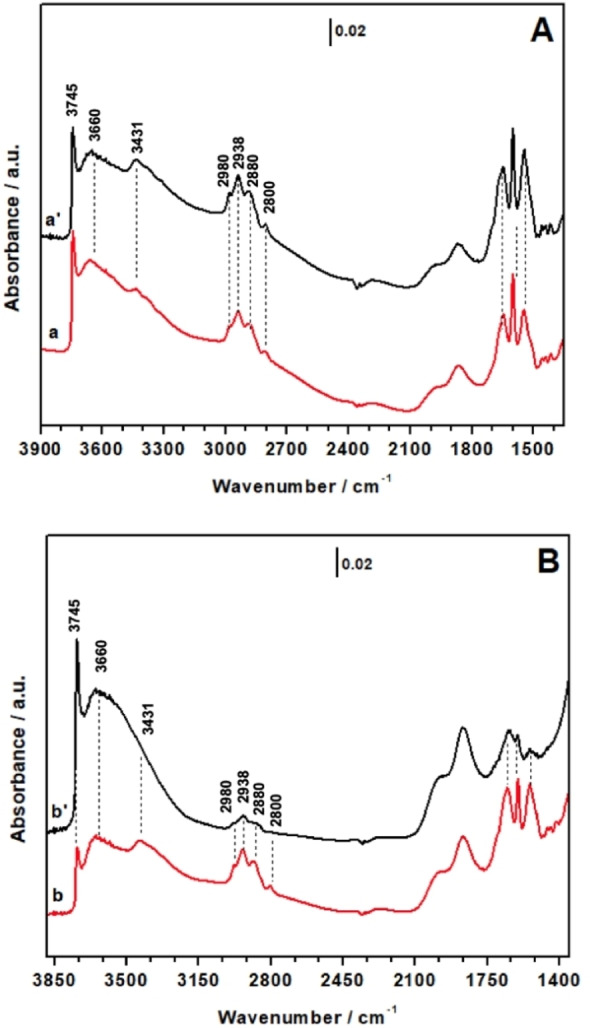
FT‐IR spectra of A) Hyb‐1 (red curve) and Hyb‐3 (black curve) and B) Hyb‐2 (red curve) and Hyb‐4 (black curve) upon outgassing the sample at 150 °C.

FT‐IR spectroscopy was used to investigate the surface vibrational modes, confirming unambiguously the presence of the organic DMAP−Si in the hybrids prepared using MeTHF as solvent (Figure 2). The FT‐IR spectra of the DMAP‐hybrids prepared using toluene have been also reported for comparison.

The FT‐IR spectra of the two hybrid materials synthesized in MeTHF show the characteristic peaks at 3745 cm^−1^ related to the stretching of the isolated silanol groups (Si‐OH) on the surface of the siliceous inorganic supports (MCM‐41NPs and Aerosil silica), followed at 3660 cm^−1^ by the signals assigned to the stretching mode of H‐bonded vicinal silanols.[^23^, ^24^] The presence of the vibrations of isolated Si‐OH groups reveals that not all silanols have been consumed by the grafting of the organic moiety. The signal due to νN−H of the secondary amine is found at 3431 cm^−1^, followed by the asymmetrical and symmetrical stretching mode of methylene groups of the organic molecule at 2938 cm^−1^ and 2880 cm^−1^, respectively. Asymmetrical and symmetrical stretching modes of methyl groups are responsible for the signals at 2980 cm^−1^ and 2800 cm^−1^, respectively. Typical signals due to overtones and combinations modes of fundamental vibrations of silica are present between 2100 cm^−1^ and 1800 cm^−1^. Signals due to urea bond (νC=O, νC−N, δN−H) and to ring stretching modes together contribute to the pattern of signal in the 1700–1500 cm^−1^ range.[^25^, ^26^] At lower frequencies (1500‐1350 cm^−1^) weak signals ascribed to deformation modes of methyl and methylene groups are visible. The presence of all these signals confirms the successful grafting and integrity of the DMAP−Si on the surface of the inorganic supports.[^27^, ^28^] It is worth noting that no signals due to isocyanate groups are present in the 2300–2000 cm^−1^ range, confirming that the alkoxysilane precursor was completely converted into the DMAP‐derivative, so that only one functionality was grafted on the silica surface. Traditional approaches, as mentioned before, include the grafting of the alkoxysilane precursor followed by the anchoring of the catalytic moiety, which result in the presence on the surface of both reacted and unreacted alkoxysilane precursors. On the contrary, our approach allows to introduce only the catalytic moiety on the surface.

**Figure 3:**
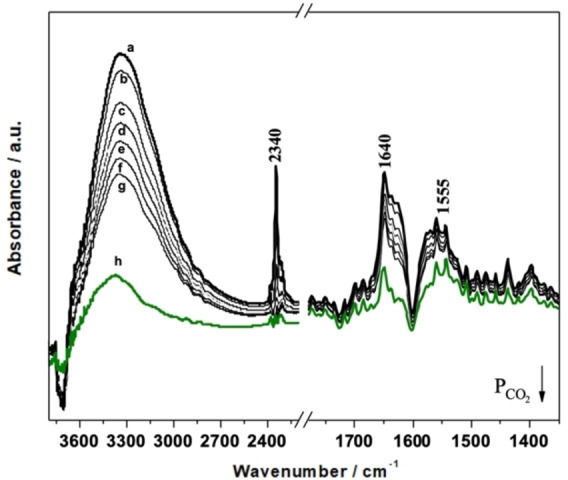
FT‐IR spectra of Hyb‐1 upon CO_2_ adsorption (max pressure: 45 mbar) and CO_2_ outgassing at room temperature.

To further evidence the presence of the DMAP−Si on silica and to assess its accessibility, CO_2_ was used as probe molecule and its adsorption at room temperature was followed by FT‐IR spectroscopy (Figure 3).

Upon adsorption of CO_2_, in the FT‐IR spectra of both hybrids (Hyb‐2 in Figure S14 in the SI) a band due to linearly adsorbed CO_2_ molecules appears at 2340 cm^−1^, which is red‐shifted with respect to the gas‐phase value (2349 cm^−1^).^[29]^ The band intensity declines by decreasing the CO_2_ pressures (curves a–g), and completely disappears upon outgassing the samples at room temperature (curves h). This band is assigned to the interaction between CO_2_ and surface Si−OH groups, in fact is also present when CO_2_ is adsorbed on pure MCM‐41NPs. The interaction of CO_2_ with silanol groups is also proved by the appearance of a negative band at ca. 3745 cm^−1^, together with a broad absorption at ca. 3300 cm^−1^. A series of positive bands in the low frequency region (below 1700 cm^−1^), which do not completely disappear upon outgassing at r.t., can be ascribed to the species formed upon interaction of CO_2_ with the NH groups of the urea group.

**Scheme 3:**
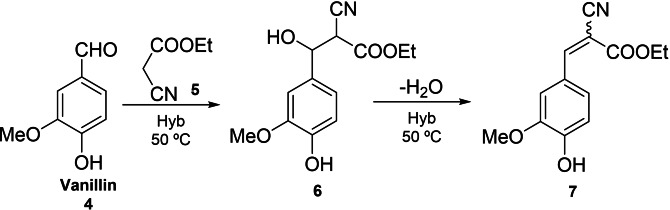
Knoevenagel condensation between vanillin and ethyl cyanoacetate under microwave heating (50 °C).

Herein the catalytic performance of DMAP‐hybrid materials (Hyb‐1 and Hyb‐2) as solid base catalysts has been evaluated for the Knoevenagel condensation between vanillin (**4**) and ethyl cyanoacetate (**5**) at 50 °C to give ethyl 2‐cyano‐3‐(4‐hydroxy‐3‐methoxyphenyl) acrylate (**7**), using MeTHF (methyl tetrahydrofuran) as green solvent (Scheme 3). Knoevenagel condensation is catalyzed by homogeneous weak bases such as primary, secondary, tertiary amines and ammonium salts.

**Figure 4:**
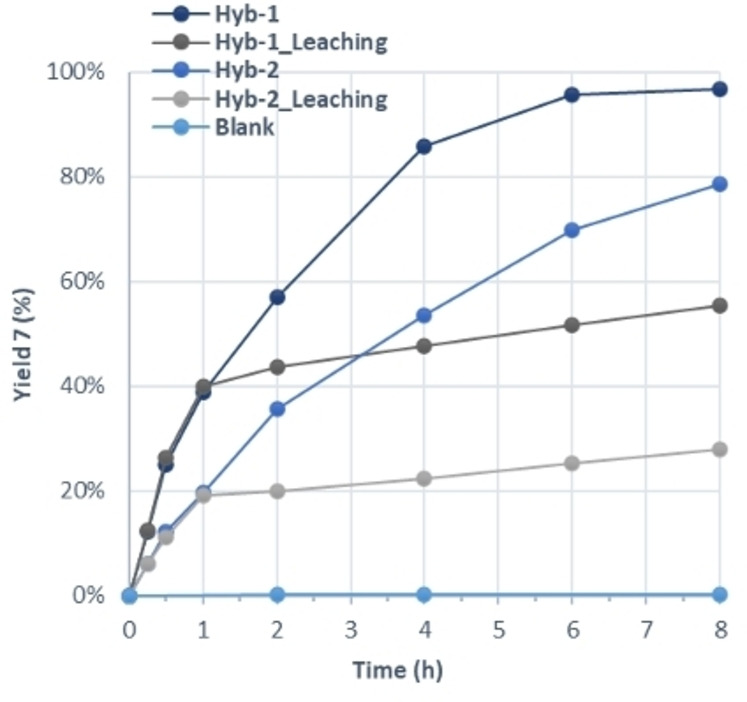
Catalytic results for the Knoevenagel condensation between vanillin (4) and ethyl cyanoacetate (5) in MeTHF at 50 °C in the presence of Hyb‐1 and Hyb‐2 catalysts (1 mol% DMAP groups).

First, the catalytic performance of Hyb‐1 and Hyb‐2 was evaluated for the Knoevenagel condensation between (**4**) and (**5**) in MeTHF at 50 °C. Before their use, hybrid catalysts underwent Soxhlet extraction for 24 h with MeTHF as solvent to ensure that all traces of not fully anchored DMAP and other organic residues were removed. As depicted in Figure 4, both samples exhibited high catalytic performance while the Hyb‐1 catalyst exhibited superior activity compared to Hyb‐2. These results can be attributed to higher surface area and mesoporous structure of the Hyb‐1 catalyst favoring both the diffusion of reactants and products of the reaction in the porous system of the solid catalyst and the accessibility to the active sites. Diffusion is a key and limiting parameter in heterogeneous catalysis. Indeed, the reactive molecules must diffuse through the reaction medium surrounding the catalyst particles (external diffusion) and then through the pore inside the particle (internal diffusion) to the active site, while the product molecule will have to diffuse out of the particle. After 8 h of time on stream (TOS), 97 % yield and 99 % selectivity of ethyl 2‐cyano‐3‐(4‐hydroxy‐3‐methoxyphenyl) acrylate (**7**) were obtained in the presence of Hyb‐1 while in the presence of Hyb‐2, 79 % yield and 99 % selectivity were reached. The notable difference in yield underscores the influence of the structural properties of Hyb‐1, which enhance mass transfer and reactivity.

Additionally, leaching tests for the Hyb‐1 and Hyb‐2 catalysts were carried out. These experiments provide insights about the activity and stability of the anchored DMAP moieties. In both cases, the catalyst was removed by filtration from the solution reaction after one hour of TOS and the process was continued for additional 8 h. Moreover, an experiment under the same reaction conditions was performed without catalyst (blank). Figure 4 displays the % yield of compound **7** versus time for these experiments. For blank, no activity was registered and <1 % yield **7** was reached after 8 h of TOS. For leaching tests, initially, in the presence of each catalyst, the conversion increases progressively, indicating effective catalytic activity, while after the removal of the catalyst (1 h), the yield of **7** increased marginally from 40 % to 55 % for Hyb‐1 and from 19 % to 28 % for Hyb‐2 (Figure 4). These findings indicated minimal catalytic contribution from potentially released active species from DMAP‐catalysts, and that the catalytic activity was primarily due to the anchored moieties, confirming the effective anchoring of the DMAP moieties and validating their role as effective heterogeneous catalysts for the Knoevenagel condensation.

**Figure 5:**
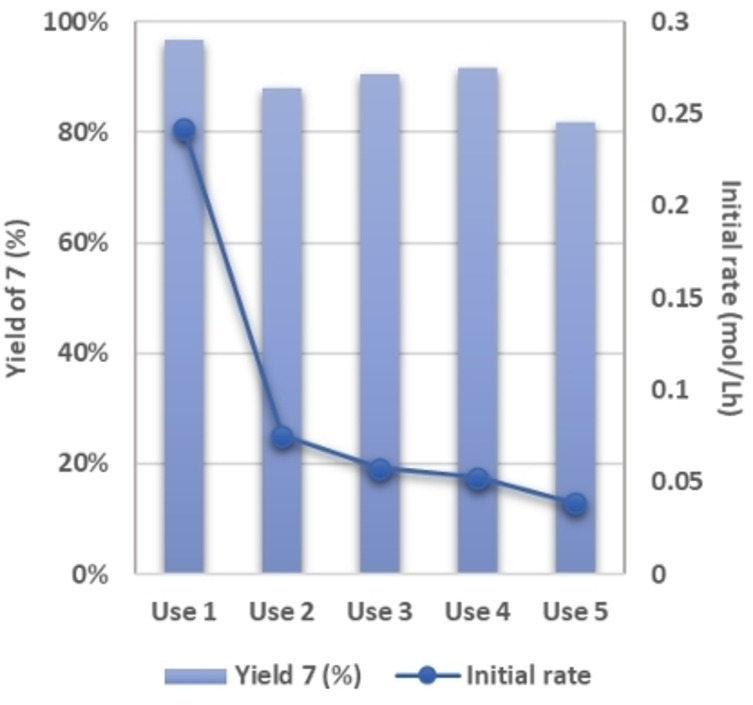
Catalytic results for the recycling study of the Hyb‐1 material as catalyst for the Knoevenagel condensation between vanillin (4) and ethyl cyanoacetate (5) in MeTHF at 50 °C (1 mol% DMAP group).

To continue and for assessing the stability and robustness of the anchored DMAP groups and their catalytic performance, a recycling study of the Hyb‐1 catalyst was conducted for the Knoevenagel condensation between ethyl cyanoacetate and vanillin with 1 mol% of DMAP groups. The catalytic results over five consecutive runs are displayed in Figure 5. While the yield of **7** remained high in the first four uses, with a decrease in the last fifth use (82 %), a pronounced decrease in the initial rate was observed after the first run. A decline in the initial rate is usually observed for heterogenous catalysts and can be ascribed to the adsorption of organic residues on the catalytic surface which can block some active sites and modifying the adsorption/desorption properties of the catalyst and diffusion of the reactants and products. All these results imply that Hyb‐1 is an active, stable, robust and reusable catalyst for the studied Knoevenagel reaction, reducing waste and improving the overall sustainability of the chemical process. Overall, these results validate the more sustainable procedure herein reported for the synthesis of functionalized hybrid catalyst involving the anchoring of the functionalized DMAP derivative on ordered mesoporous or amorphous silica in readily available, inexpensive and biobased solvent, MeTHF.

Finally, a short study of the catalytic performance of Hyb‐1 for the Knoevenagel condensation between ethyl cyanoacetate and aromatic aldehydes with various substituent groups such as nitro, methyl, methoxyl and hydroxyl was carried out (Table 3). Good to excellent yields (87.5–99 %) were obtained in 6–8 h reaction time. Electron‐rich aldehydes (anisaldehyde or tolualdehyde) achieved good yields of up to 88 % (entries 1–2) under the reaction conditions. Reactions of ethyl 2‐cyanoacetate with electron‐deficient 4‐nitrobenzaldehyde, syringaldehyde and vanillin gave excellent yields of up to 99 % (entries 3–5). In contrast, when ethyl acetoacetate, a low‐activation methylene compound, was reacted with vanillin, Knoevenagel condensation did not occur (entry 6).

**Table 3:** Hyb‐1 scope for catalysed Knoevenagel condensation between benzaldehyde derivatives and activated esters.

Entry	Benzaldehyde derivatives	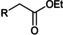		Yield % (initial rate)	Time (h)
1	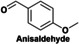	R=CN		87.5	8
				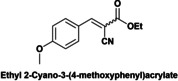	
2	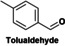	R=CN		88	8
				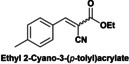	
3	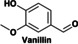	R=CN		97	8
				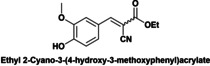	
4	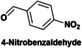	R=CN		99	5
				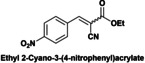	
5	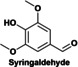	R=CN		90.5	6
				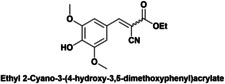	
6	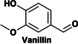	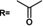		–	24
				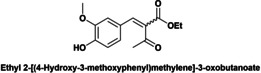	

Reaction conditions: 2 mL MeTHF, 50 °C, 75 mg of Hyb‐1, 1 mmol of benzaldehyde derivative and 1.2 mmol active methylene compound (RCOOEt).

## Conclusions

In conclusion, this work highlights a sustainable strategy for the functionalization of the DMAP molecule to create an effective and recyclable hybrid catalyst. By introducing for the first time a reactive aminomethyl group on the DMAP nucleus, the active molecule was successfully and stably anchored onto mesoporous MCM‐41 NPs and Aerosil silica supports, enabling its use in heterogeneous catalysis while avoiding the environmental risks associated with DMAP's toxicity. This functionalization approach overcomes challenges in modifying the DMAP structure, providing a robust covalent linkage that enhances stability and allows efficient recovery and reuse. The synthesized hybrids were characterized through an array of physico‐chemical techniques, including X‐ray diffraction (XRD), thermogravimetric analysis (TGA), Fourier‐transform infrared (FT‐IR) spectroscopy, and nitrogen adsorption/desorption analysis. These analyses confirmed the structural integrity and thermal stability of the hybrid materials, revealing that the ordered mesoporous structure of MCM‐41 was preserved post‐grafting and that the functionalized DMAP was effectively incorporated onto the silica surfaces. Additionally, monitoring CO₂ adsorption by FT‐IR provided insights into surface interactions and active site accessibility, confirming the hybrid's suitability for catalytic applications. The modified DMAP hybrids demonstrated excellent catalytic performance for the Knoevenagel condensation reaction, particularly when supported on MCM‐41 NPs, which enhanced mass transfer due to its high surface area and pore structure. The use of MeTHF as a renewable solvent further underscores the eco‐friendly nature of this approach, minimizing reliance on nonrenewable, petroleum‐based solvents. Overall, the successful functionalization and immobilization of DMAP on silica support materials not only advance the sustainability of catalytic processes but also present a versatile platform for green chemistry applications in organic synthesis.

## Experimental Section

### Materials

Solvents and starting materials were purchased from Merck (Milano, Italy) or TCI Europe (Zwijndrecht, Belgium) and used without further purification. All aqueous solutions were prepared from ultrapure laboratory grade water (18 MΩ・cm) obtained from Milli‐Q ultrapure water system (Millipore, Burlington, Massachussets, USA).

### Synthetic Procedures

**4‐(*N,N*‐Dimethylamino)‐3‐phthalimidomethylpyridin**e (**1**). DMAP (15.0 g, 123 mmol, 1.00 eq) was added in small portions to sulphuric acid (80 mL) at 0 °C (caution: strongly exothermic reaction). Phthalimide (21.70 g, 147 mmol, 1.20 eq) and paraformaldehyde (5.53 g, 184 mmol, 1.50 eq) were added and the mixture was heated at 70 °C for 5 hours. After complete conversion (TLC: DCM/MeOH/conc. NH_4_OH 92/8/0.2), the mixture was cautiously poured in cold water and slowly basified to pH=10 by careful addition of Na_2_CO_3_. Compound 1 separated as a white solid and was isolated by filtration under vacuum, washed with distilled water and dried in under vacuum to constant weight (yield 94 %, 32.5 g).

^1^H NMR (301 MHz, CDCl_3_, 298 K) δ 8.25 (d, J=5.6 Hz, 1H), 8.11 (s, 1H), 7.81 (dd, J=5.5/3.1 Hz, 2H), 7.68 (dd, J=5.5/3.1 Hz, 2H), 6.78 (d, J=5.5 Hz, 1H), 4.87 (s, 2H), 2.82 (s, 6H) ppm.

^13^C NMR (76 MHz, CDCl_3_, 298 K) δ 168.0 (C), 157.4 (C), 149.4 (CH), 148.9 (CH), 134.2 (CH), 132.0 (C), 123.4 (CH), 123.1 (C), 112.4 (CH), 43.4 (CH_3_), 36.3 (CH_2_) ppm.

MS (ESI^+^): m/z=304.10 ([M+Na]^+^), 282.12 ([M+H]^+^). Calc. For C_16_H_15_N_3_O_2_+Na^+^: 304.11 and C_16_H_15_N_3_O_2_+H^+^: 282.12.

HRMS (ESI^+^): m/z=282.12350 ([M+H]^+^). Calc. For C_16_H_15_N_3_O_2_+H^+^: 282.12370.

**3‐Aminomethyl‐4‐(*N,N*‐Dimethylamino)pyridine** (**2**). Compound **1** (30.0 g, 107 mmol, 1.00 eq) was suspended in ethanol (250 mL), hydrazine hydrate (6.66 mL, 149 mmol, 1.4 eq) was added and the mixture was refluxed for 4 h. After complete deprotection (TLC: DCM/MeOH/conc. NH_4_OH 92/8/0.2), the mixture was cooled with an ice bath and the precipitate was filtered under vacuum. The solid was dissolved in 120 mL of warm NaOH 30 % and extracted with toluene (6x50 mL). The pooled organic phases were dried over sodium carbonate, filtered and concentrated under vacuum to give the product as a yellow oil (yield 74 %, 12.0 g).

^1^H NMR (400 MHz, CDCl_3_, 298 K) δ 8.25 (s, 1H), 8.17 (d, J=5.6 Hz, 1H), 6.64 (d, J=5.6 Hz, 1H), 3.81 (s, 2H), 2.77 (s, 6H), 1.83 (br s, 2H) ppm.

^13^C NMR (101 MHz, CDCl_3_, 298 K) δ 157.5 (C), 150.4 (CH), 148.9 (CH), 128.4 (C), 111.8 (CH), 42.7 (CH_3_), 42.0 (CH_2_) ppm.

HRMS (ESI^+^): m/z=152.11824 (100 %, [M+H]^+^), 135.09171 (15 %). Calc. For C_8_H_13_N_3_+H^+^: 152.11822.

**1‐((4‐(*N,N*‐Dimethylamino)pyridin‐3‐yl)methyl)‐3‐(3‐(triethoxysilyl)propyl)urea** (**3**). Compound **2** (1.00 g, 6.61 mmol, 1.00 eq) was dissolved under nitrogen atmosphere in dry DCM (10 mL). 3‐(triethoxysilyl)propyl isocyanate (1.72 g, 6.95 mmol, 1.05 eq) was added in one portion to the mixture. The resulting solution was left at RT for 15 minutes, monitoring the reaction by TLC analysis (DCM/MeOH 8 : 2). The reaction mixture was concentrated under vacuum to give the product as a white solid (yield 96 %, 2.53 g).

^1^H NMR (400 MHz, CDCl_3_, 298 K) δ 8.20 (s, 1H), 8.12 (d, J=5.6 Hz, 1H), 6.62 (d, J=5.7 Hz, 1H), 5.63 (br t, J=5.2 Hz, 2H), 4.31 (d, J=4.5 Hz, 2H), 3.72 (q, J=7.0 Hz, 6H), 3.09 (t, J=7.2 Hz, 2H), 2.76 (s, 6H), 1.52 (p, J=8.3 Hz, 2H), 1.13 (t, J=7.0 Hz, 9H), 0.54 (t, J=8.4 Hz, 2H) ppm.

^13^C NMR (101 MHz, CDCl_3_, 298 K) δ 158.7 (C), 157.8 (C), 150.2 (CH), 148.6 (CH), 125.0 (C), 111.6 (CH), 58.4 (CH_2_), 42.9 (CH_2_), 42.8 (CH_3_), 39.6 (CH_2_), 23.7 (CH_2_), 18.3 (CH_3_), 7.6 (CH_2_) ppm.

HRMS (ESI^+^): m/z=399.24246 ([M+H]^+^). Calc. For C_18_H_34_N_4_O_4_Si+H^+^: 399. 24221.

**Hyb_1‐4**. The grafting reaction was carried out by dissolving 30.0 mg (0.0753 mmol) of compound **3** in 19 mL of solvent (2‐methyltetrahydrofuran or toluene). To this mixture 300 mg (loading=10 % by weight) of the inorganic support (MCM‐41 or Aerosil) were added. This suspension was stirred at 60 °C for 24 hours. The material was recovered by vacuum filtration, washed with the reaction solvent (2‐methyltetrahydrofuran or toluene), dried in an oven at 80 °C for 8 h and analysed by FT‐IR spectroscopy.

### Characterization Techniques

#### Characterization of the DMAP‐Derivatives

NMR spectra were recorded at RT, at 300 MHz on a Jeol Eclipse ECP300 spectrometer (Jeol Ltd., tokyp, Japan) or at 400 MHz on a Bruker Avance Neo 400 spectrometer (Milano, Italy). Chemical shifts (δ) are quoted to parts per million referenced to the residual solvent peak. The multiplicity of each signal is designated using the following abbreviations: s, singlet; d, doublet; t, triplet; q, quartet; p, quintet; sext, sextet; sept, septet; m, multiplet, br s, broad singlet; br m, broad multiplet. Coupling constants (J) are reported in Hertz (Hz). NMR spectra are reported in the ESI (Figures S1‐S6). Mass spectra were obtained with a TSQ Quantum Access Max Triple Quadrupole Mass Spectrometer (Thermo Scientific, Monza, Italy) equipped with an electrospray ionization (ESI) source, analysis by direct infusion. Mass spectra are reported in the ESI (Figures S7, S8 and S9). TLC were performed with silica gel (MN Kieselgel 60F254, Merck, Milano, Italy) and visualized by UV or sprayed with Dragendorff reagent or alkaline KMnO_4_.

#### Characterization of the Hybrids

All hybrids were characterized by X‐ray powder diffraction (XRPD), field emission scanning electron microscopy (FE‐SEM), thermogravimetric analysis (TGA), and FT‐IR spectroscopies using probe molecules to show the functionalities of the hybrid catalyst. Moreover, the mesoporous Hyb‐1 was characterized by N_2_ adsorption/desorption at 77 K.

XRPD patterns were obtained using a Bruker AXS D8 ADVANCE diffractometer (Karlsruhe, Germany), in reflection mode with Bragg–Brentano geometry, operating with a radiation source of monochromatic X‐rays Cu Kα (λ=1.5406 Å) and an LYNXEYE_XE_T high‐resolution position‐sensitive detector. XRPD patterns were recorded in the 0.5–10° and 5–60° (2θ) range at the voltage and amperage of the source 40 kV/40 mA, with a coupled 2θ‐θ method, at a scan speed of 0.100 s/step and a step size of 0.01° [Figure S10 in the ESI].

FE‐SEM analysis was performed using a GeminiSEM‐360 instrument (Carl Zeiss S.p.A., Milan, Italy). Samples were Pt‐sputter‐coated (20 nm thick Pt layer) under argon (Emitech K575X Turbo Pumped Sputter Coater, Quorum Technologies, UK).

TGA/DTA were carried out in air stream (60 mL/min) using a Setaram LABSYS evo instrument (Caluire, France), heating from 30 °C to 800 °C at 5 °C/min.

FT‐IR spectra were obtained using a Nicolet 5700 (Thermo Fisher Scientific, Monza, Italy) spectrometer equipped with DTGS detector. Spectra were acquired on self‐supporting pellets at a resolution of 4 cm^−1^ in a 4000–400 cm^−1^ spectral range, over 64 scans. All spectra were collected after a thermal treatment of the hybrids at 150 °C for 1 h under vacuum conditions (residual pressure <10^−4^ mbar). FT‐IR spectra were normalized with respect to the pellet density. The FT‐IR spectroscopic study was implemented through the aid of probe molecules capable of detecting the presence of active sites on the different synthesized hybrids. In particular, the basic capacity of the DMAP was detected using CO_2_ as probe molecule.

### Catalytic Tests

The catalytic performances of the DMAP‐hybrid catalysts were tested for the Knoevenagel condensation. The experiments were performed under microwave heating in a Biotage® Initiator+ apparatus (Uppsala, Sweden). For a typical experiment, the desired amount of hybrid catalyst (1 mol% DMAP group, 50 mg), 1 mmol of vanillin and 1.2 mmol of ethyl cyanoacetate were added to 2 mL of MeTHF in a microwave reaction vial of 5 mL. The vial was sealed, and the reaction medium was heated to the desired temperature for a given time. Quantification of conversion, yield and selectivity was made by monitoring the reaction at different time through GC‐FID analysis (Shimadzu, GC2030, equipped with a HP‐5 column (0.25 μm*0.25 mm*30 m) and GC‐MS analysis (Shimadzu, GCMS‐QP2010 Ultra). For the scope study, 75 mg (1.5 mol% DMAP group) of hybrid catalyst, 1 mmol of benzaldehyde derivatives and 1.2 mmol of active methylene group were added to 2 mL of MeTHF in a microwave reaction vial of 5 mL. For the recycling study, the catalyst was filtered and washed extensively with acetone, dried overnight in an oven at 100 °C and used for the next run.

## Conflict of Interests

The authors declare no conflict of interest.

1

## Supporting information

As a service to our authors and readers, this journal provides supporting information supplied by the authors. Such materials are peer reviewed and may be re‐organized for online delivery, but are not copy‐edited or typeset. Technical support issues arising from supporting information (other than missing files) should be addressed to the authors.

Supporting Information

## Data Availability

The data that support the findings of this study are available from the corresponding author upon reasonable request.
