# Supplementary material for: Novel DMAP@Mesoporous Silica Hybrid Heterogeneous Catalysts for the Knoevenagel Condensation: Greener Synthesis through Eco‐friendly Solvents
Source: Chempluschem. 2025 Mar 1;90(5):e202400741. doi: 10.1002/cplu.202400741 (PMC12105418; doi:10.1002/cplu.202400741)
Supplement: Supplementary file 1 — Supporting Information [file CPLU-90-e202400741-s001.pdf]

ChemPlusChem

Supporting Information

Novel DMAP@Mesoporous Silica Hybrid Heterogeneous Catalysts for the Knoevenagel Condensation: Greener Synthesis through Eco-friendly Solvents

Julio C. Fernandes P. Brito, Fabio Travagin, Mauro Barbero, Cristina Esteban, Urbano Díaz, Alexandra Velty, Giovanni B. Giovenzana, Ivana Miletto,* and Enrica Gianotti*

- **Table of Contents**

NMR Spectra (Figures S1-S6)	2
High Resolution Mass Spectra (Figures S7-S9)	5
XRPD patterns (Figure S10)	6
N_2 adsorption/desorption isotherm (Figure S11)	7
Representative FE-SEM images (Figure S12)	7
TGA/dTA curves (Figure S13)	8
CO_2 adsorption on Hyb-2 monitored by FTIR spectroscopy (Figure S14)	8
GC-MS spectra, Knoevenagel condensation products (Figures S15-S19)	9

NMR Spectra

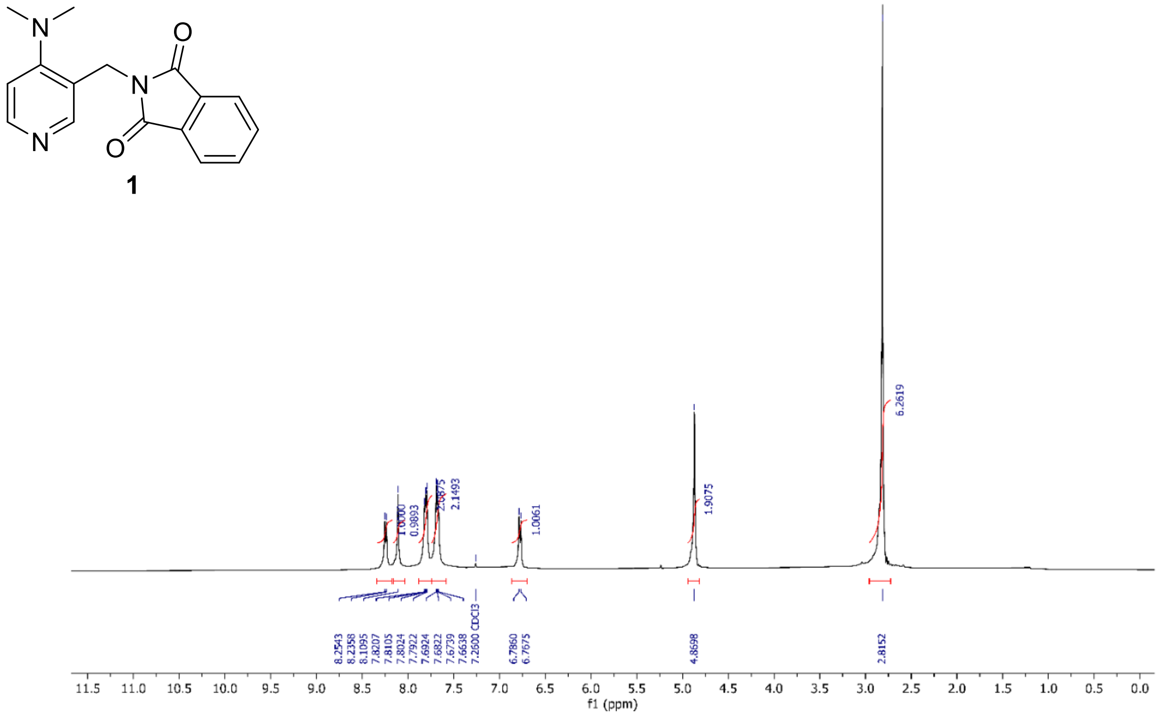

Figure S1. ^{1}H NMR spectrum of compound **1**.

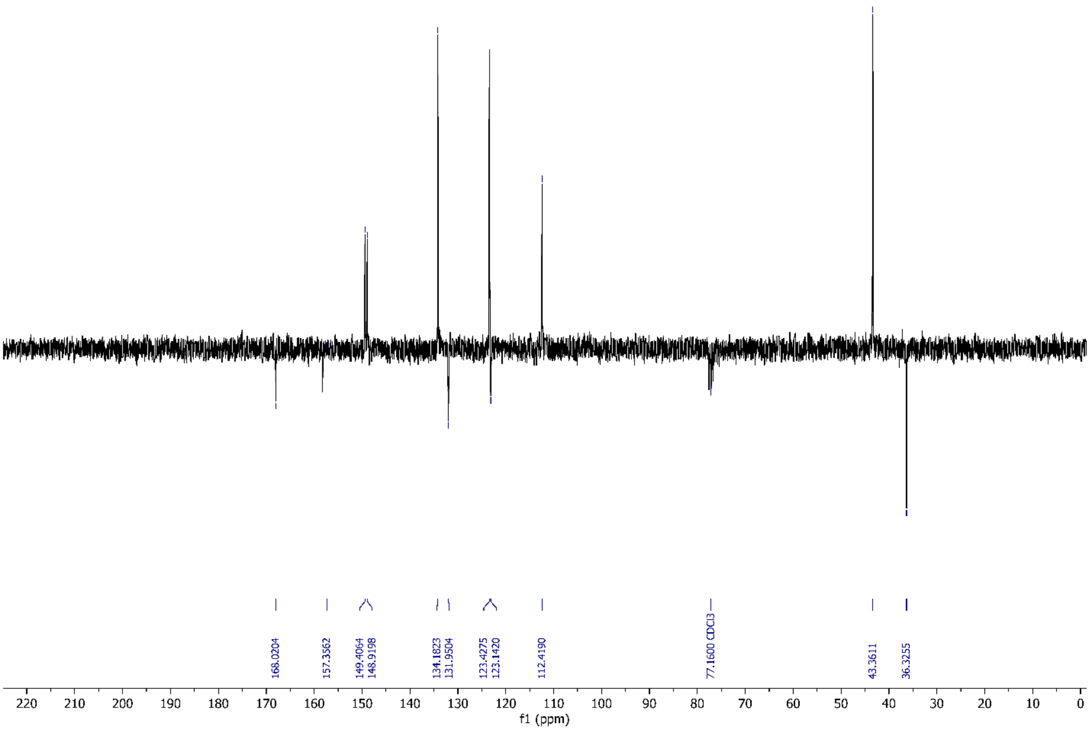

Figure S2. ^{13}C APT NMR spectrum of compound **1**.

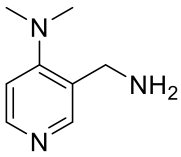

2

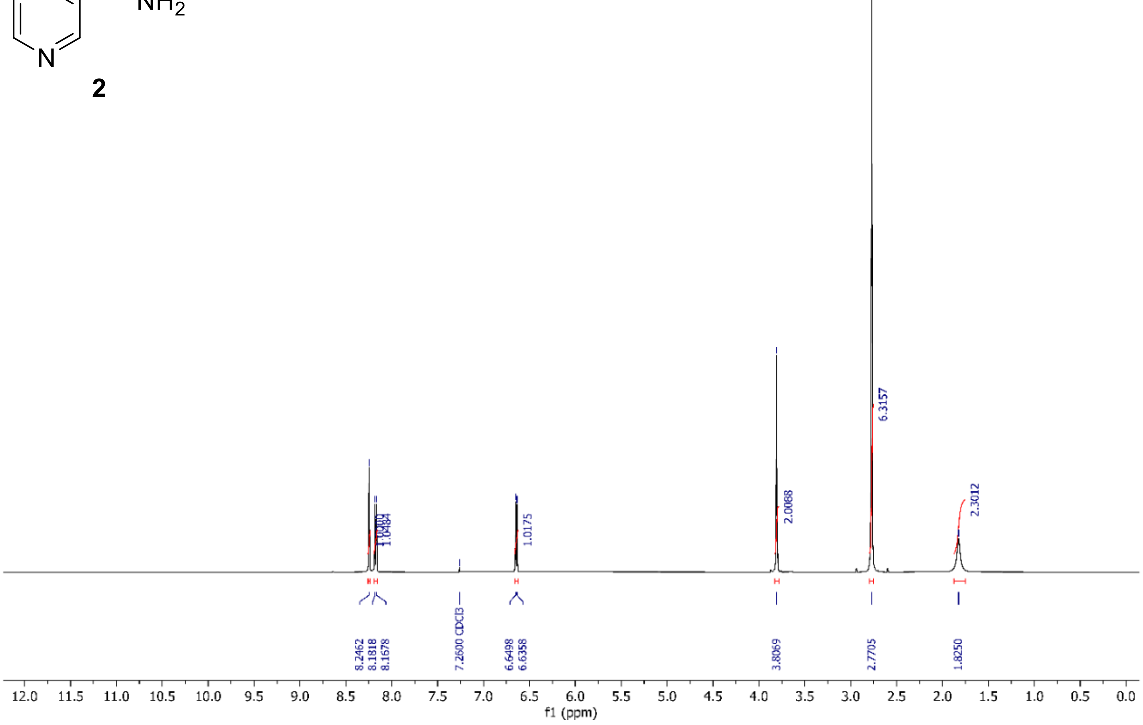

Figure S3. ^{1}H NMR spectrum of compound **2**.

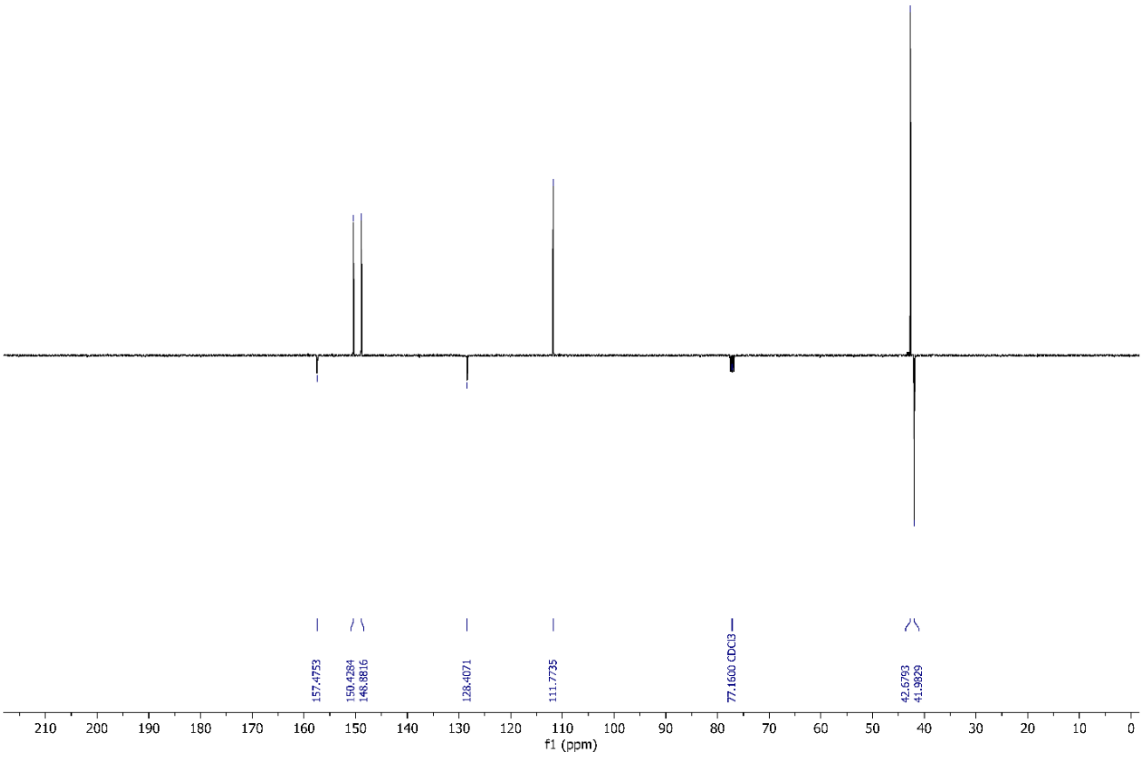

Figure S4. ^{13}C APT NMR spectrum of compound **2**.

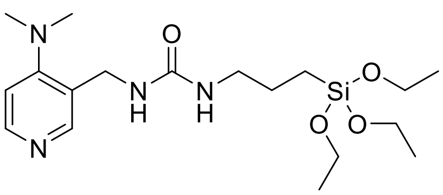

3

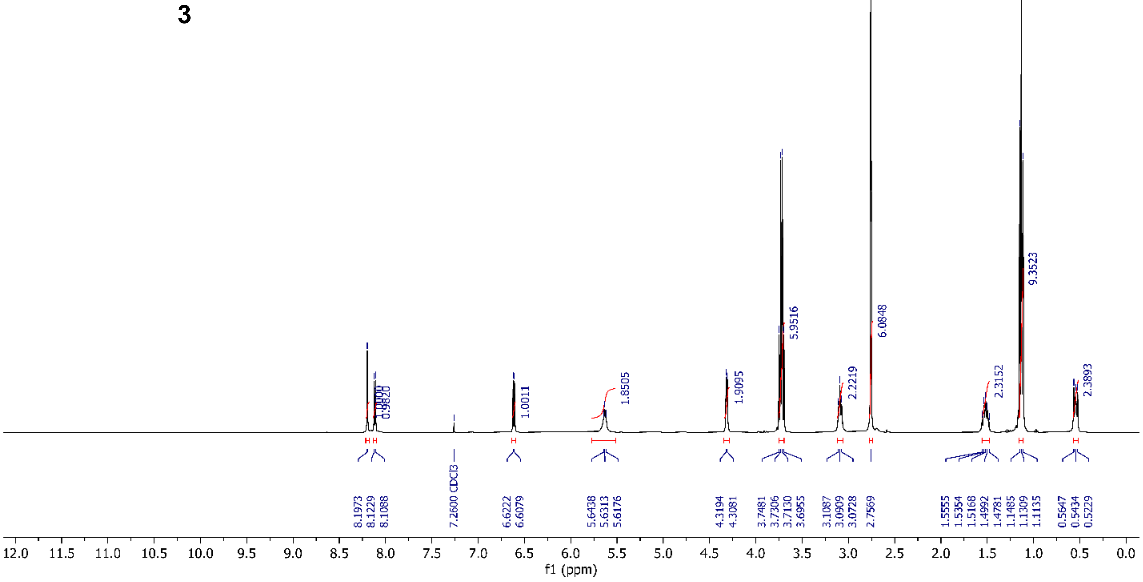

Figure S5. ^{1}H NMR spectrum of compound **3**.

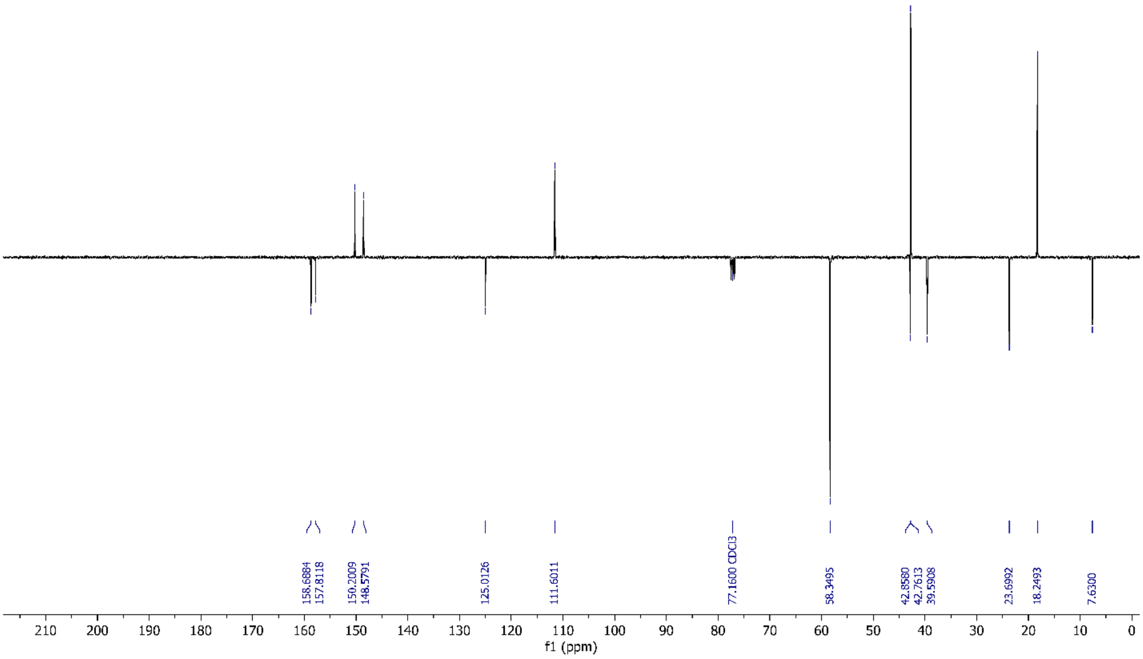

Figure S6. ^{13}C APT NMR spectrum of compound **3**.

High Resolution Mass Spectra

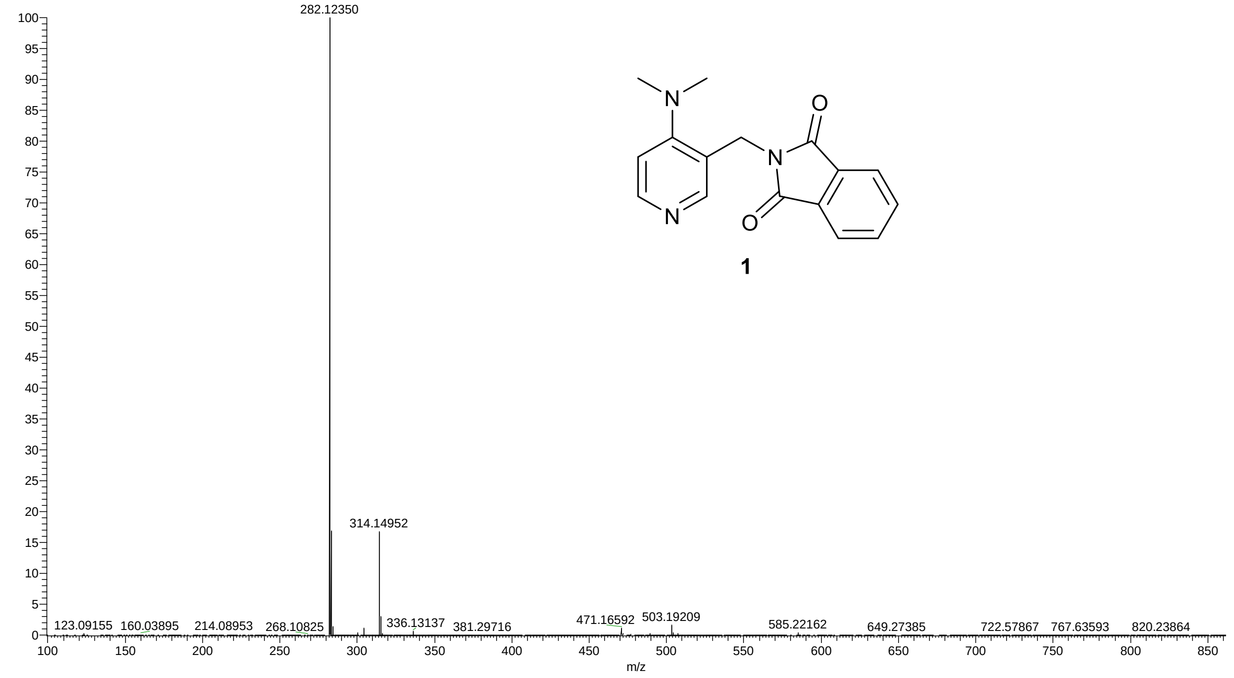

Figure S7. High resolution mass spectrum of compound **1**.

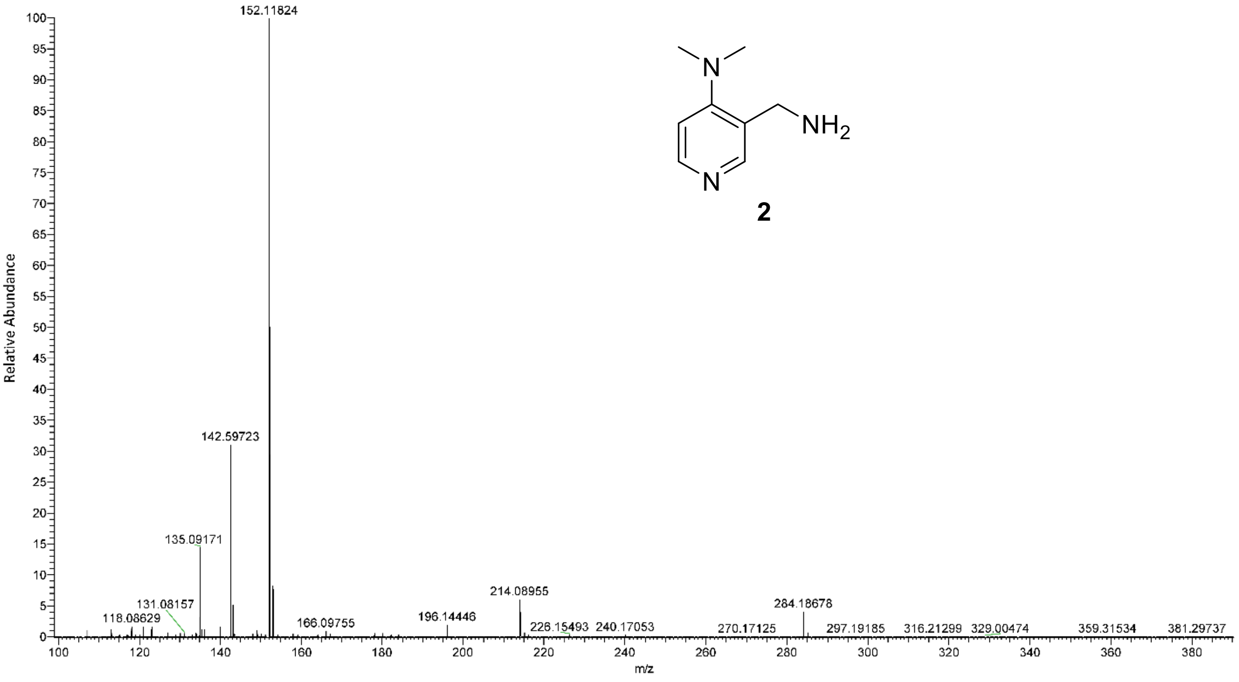

Figure S8. High resolution mass spectrum of compound **2**.

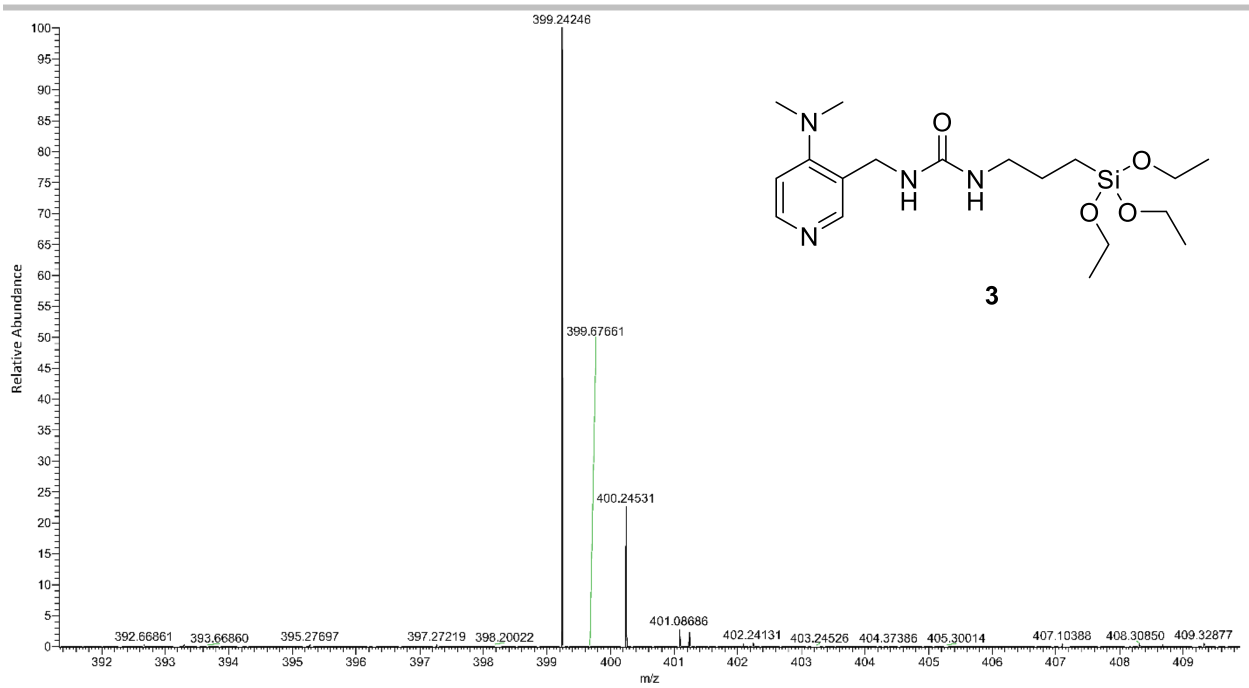

Figure S9. High resolution mass spectrum of compound **3**.

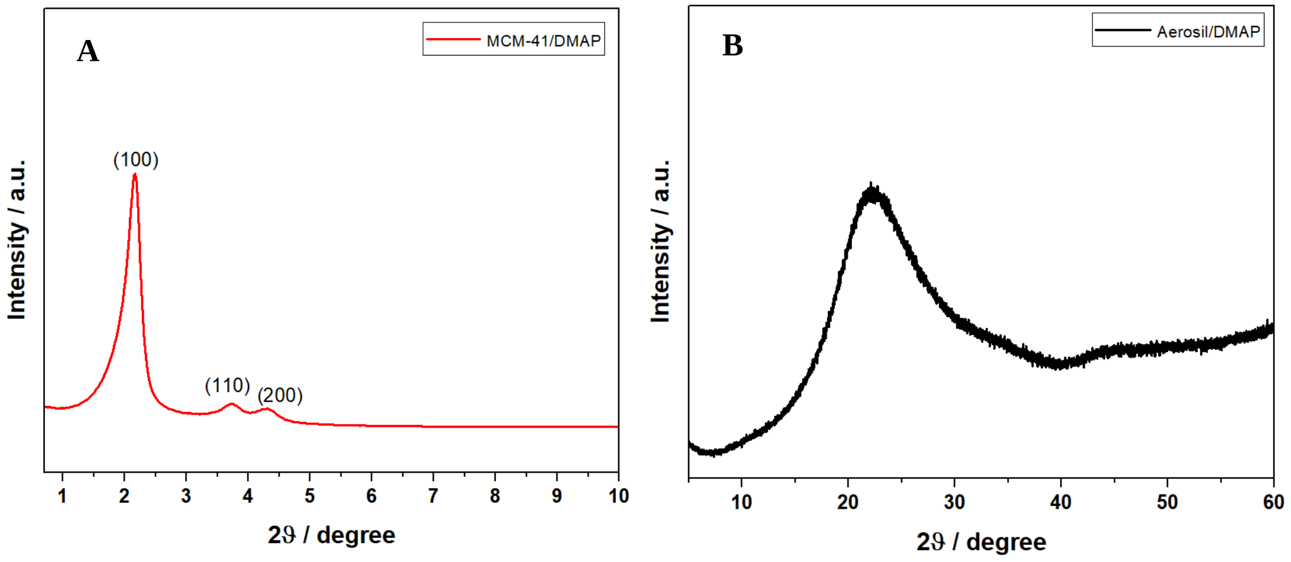

Figure S10 – XRPD diffraction patterns of MCM-41/DMAP (section A) and Aerosil/DMAP (section B).

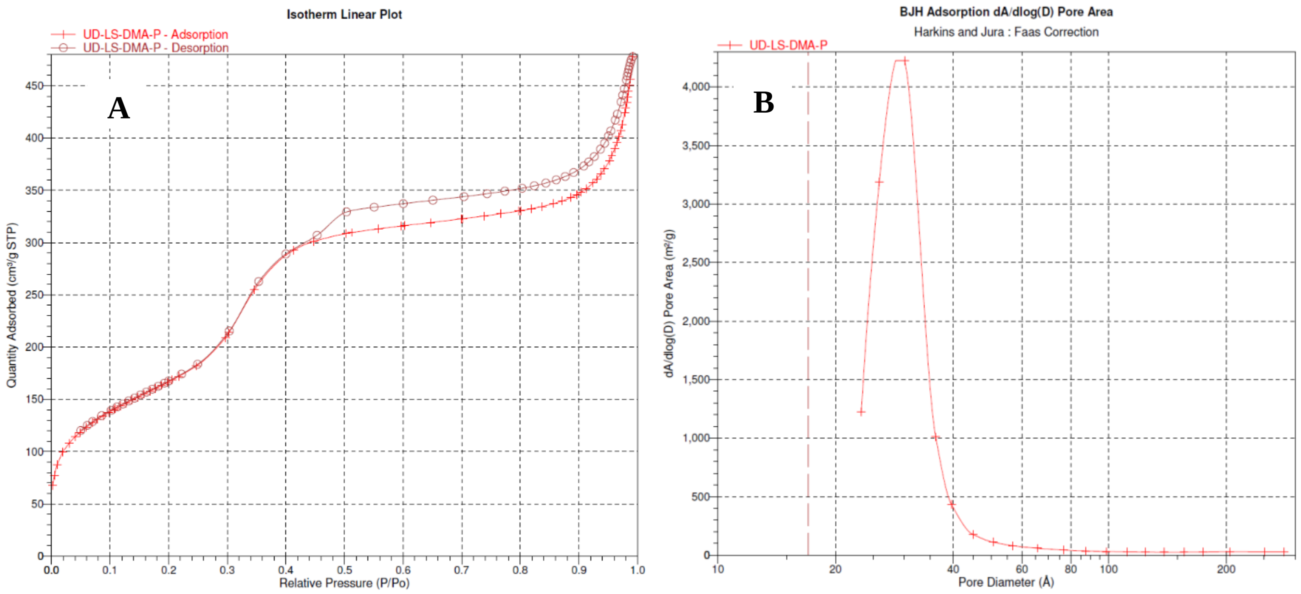

Figure S11 – N_2 adsorption/desorption isotherms at 77K (section A) and pore size distribution (section B) of MCM-41/DMAP

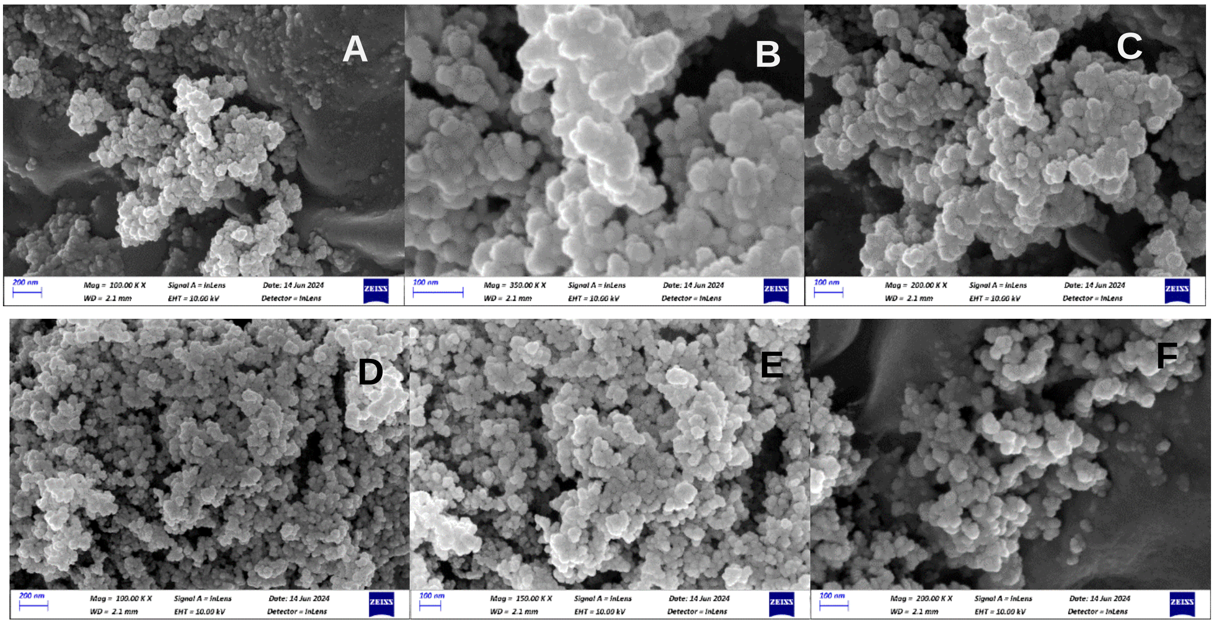

Figure S12 – Field emission-SEM images of MCM-41/DMAP (section A, B, C) and Aerosil/DMAP (section D, E, F)

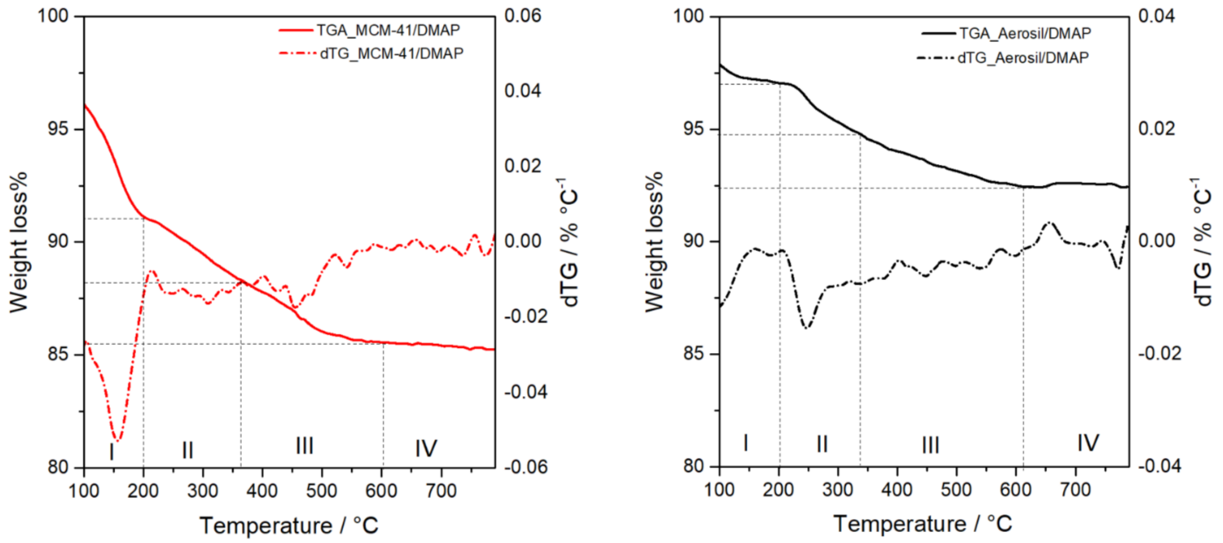

Figure S13 – TGA/dTG of MCM-41/DMAP (section A) and Aerosil/DMAP (section B) divided in four zones according to the weight range loss.

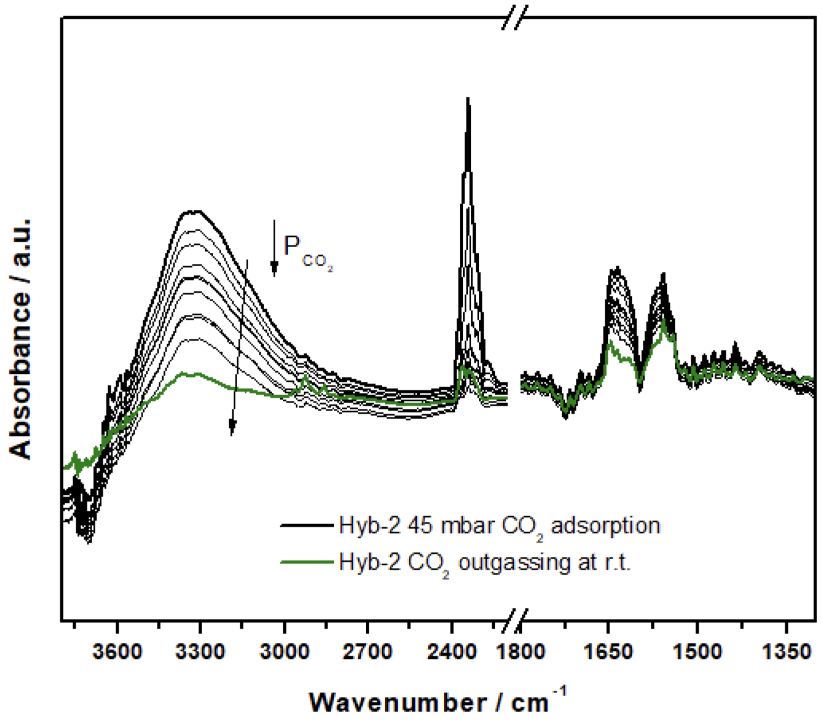

Figure S14 – FTIR spectra of Hyb-2 upon CO_2 adsorption (max pressure: 45 mbar) and CO_2 outgassing at room temperature.

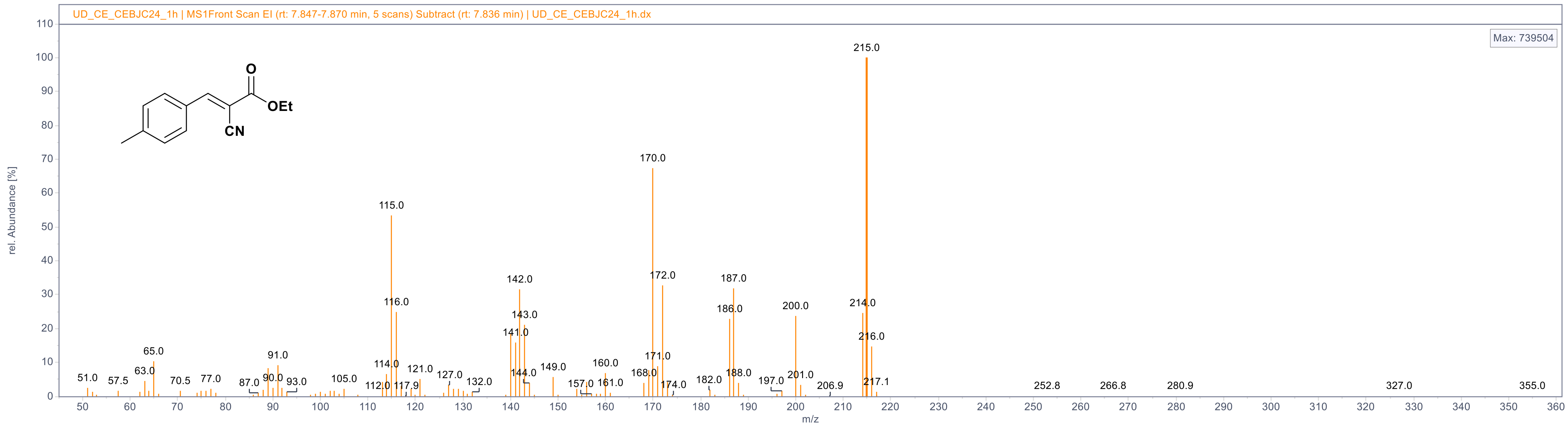

Figure S15. Mass spectrum ethyl 2-cyano-3-(p-tolyl)acrylate**.**

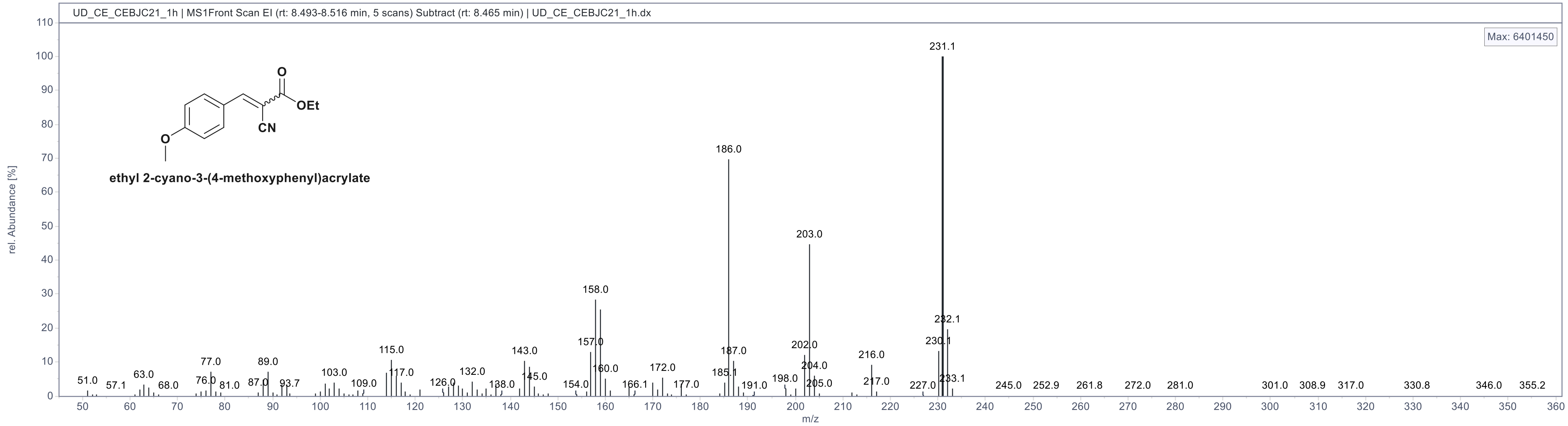

Figure S16. Mass spectrum ethyl 2-cyano-3-(4-methoxyphenyl)acrylate

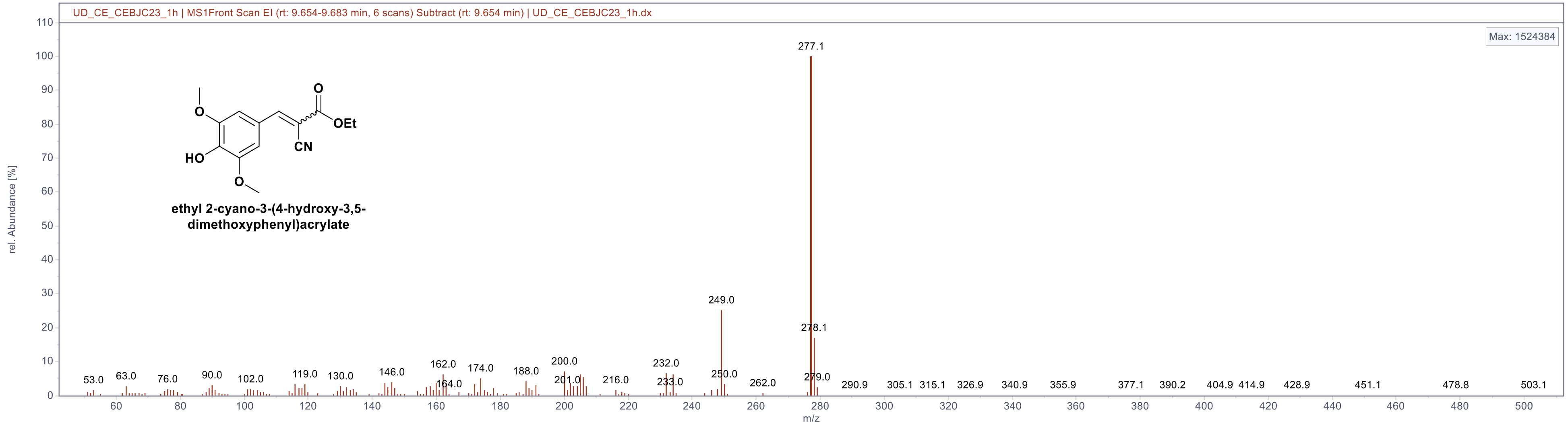

Figure S17. Mass spectrum ethyl 2-cyano-3-(4-hydroxy-3,5-dimethoxyphenyl)acrylate.

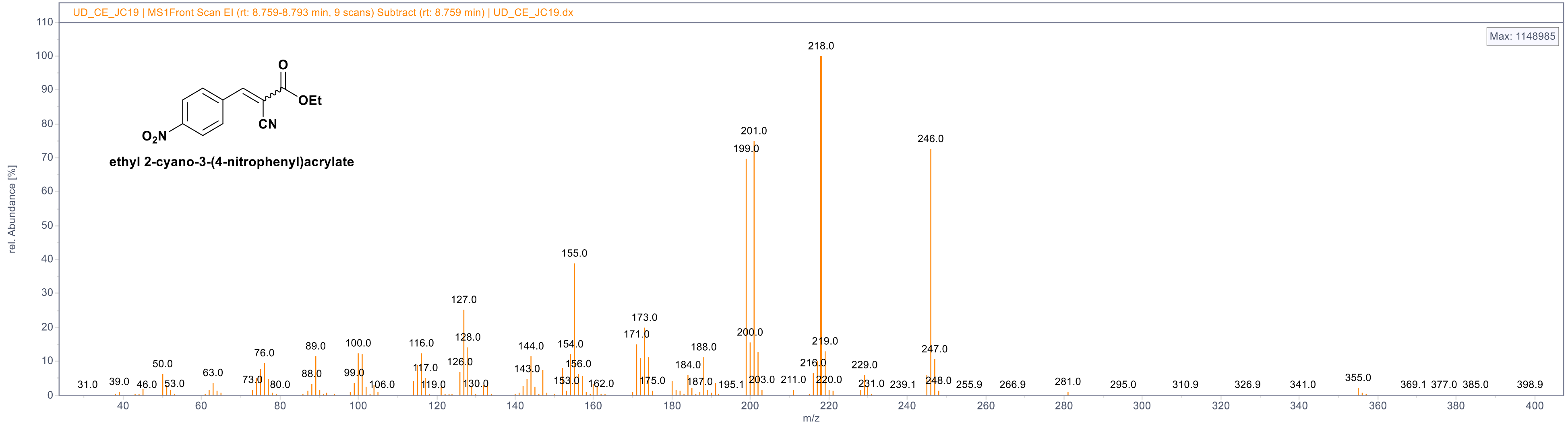

Figure S18. Mass spectrum ethyl 2-cyano-3-(4-nitrophenyl)acrylate.

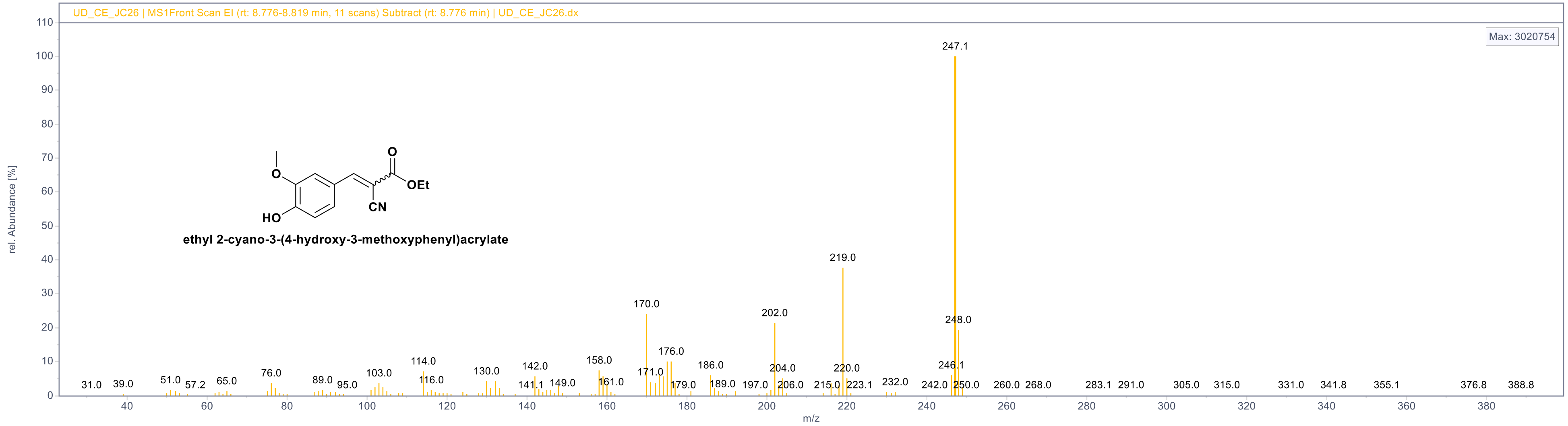

Figure S19. Mass spectrum ethyl 2-cyano-3-(4-hydroxy-3-methoxyphenyl)acrylate.
